# Signal transducer and activator of transcription (STAT)-3 regulates microRNA gene expression in chronic lymphocytic leukemia cells

**DOI:** 10.1186/1476-4598-12-50

**Published:** 2013-06-01

**Authors:** Uri Rozovski, George A Calin, Tetsuro Setoyama, Lucilla D’Abundo, David M Harris, Ping Li, Zhiming Liu, Srdana Grgurevic, Alessandra Ferrajoli, Stefan Faderl, Jan A Burger, Susan O’Brien, William G Wierda, Michael J Keating, Zeev Estrov

**Affiliations:** 1 Department of Leukemia, The University of Texas MD Anderson Cancer Center, 1515 Holcombe Blvd, Houston, TX 77030, USA; 2 Department of Experimental Therapeutics, The University of Texas MD Anderson Cancer Center, 1515 Holcombe Blvd, Houston, TX 77030, USA

**Keywords:** CLL, microRNA, STAT3

## Abstract

**Backgrounds:**

Approximately 1,000 microRNAs (miRs) are present in the human genome; however, little is known about the regulation of miR transcription. Because miR levels are deregulated in chronic lymphocytic leukemia (CLL) and signal transducer and activator of transcription (STAT)-3 is constitutively activated in CLL, we sought to determine whether STAT3 affects the transcription of miR genes in CLL cells.

**Methods:**

We used publically available data from the ENCODE project to identify putative STAT3 binding sites in the promoters of miR genes. Then we transfected CLL cells with STAT3-shRNA or with an empty vector, and to determine which miRs are differentially expressed, we used a miR microarray approach followed by validation of the microarray results for 6 miRs using quantitative real-time polymerase chain reaction (qRT-PCR).

**Results:**

We identified putative STAT3 binding sites in 160 promoter regions of 200 miRs, including miR-21, miR-29, and miR-155, whose levels have been reported to be upregulated in CLL. Levels of 72 miRs were downregulated (n = 63) or upregulated (n = 9). qRT-PCR confirmed the array data in 5 of 6 miRs.

**Conclusions:**

The presence of activated STAT3 has a profound effect on miR expression in CLL cells.

## Introduction

B-cell chronic lymphocytic leukemia (CLL) is characterized by asymmetrical proliferation and apoptosis of leukemia cells co-expressing the CD5 and CD19 antigens [1,2], and several chromosomal abnormalities, including del(13q), del(11q), del(17p), and trisomy 12, are detected in most, but not all, cases. However, in almost all patients, regardless of their cytogenetic abnormalities, clinical characteristics, disease stage, or treatment status, signal transducer and activator of transcription 3 (STAT3) is constitutively phosphorylated on serine 727 residue [3,4]. Phosphoserine STAT3 shuttles to the nucleus, binds to DNA, and activates genes known to be activated by phosphotyrosine STAT3 in other cell types [4]. Furthermore, unphosphorylated STAT3, detected at high levels in CLL cells, constitutively activates the transcription factor nuclear factor κB [5], which is known to induce the production of several pro-inflammatory cytokines and activate survival pathways.

STAT3-induced transcription of protein-coding genes has been extensively studied in normal and neoplastic tissues[6]. However, protein-coding genes comprise only 3% of the human genome [7] and only scant data are available on the role of STAT3 in the transcription of non-protein-coding genes. Although approximately 1000 microRNAs (miRs) collectively regulate more than 30% of protein-coding genes [8], little is known about miR gene transcription. Iliopoulos et al. found that STAT3 activates the transcription of miR-21 and miR-181b-1, thereby inducing a stable transformed state in cancer cell lines [9].

Because STAT3 is constitutively activated in CLL cells and recent data demonstrated a global deregulation of the miR network in CLL, [10] we hypothesized that STAT3 affects the expression of miRs in CLL cells. Therefore, we analyzed publicly available data to determine whether STAT3 binds to miR promoters, and studies the effect of STAT3 on miR expression in CLL cells.

## Methods

### Data mining

To find the putative promoter sites of miR genes, we used data on H2K4me3 enrichment regions published by Baer et al. [11]. To find STAT3 binding sites on these promoters, we used chromatin immunoprecipitation followed by high-throughput DNA sequencing (ChIP-seq) data (Additional file 1: Figure S1), generated as part of the ENCODE project [12] and acquired from the University of California Santa Cruz genome browser. For each putative STAT3 binding event a cluster score representing the ChIP-seq signal strength, ranging from 0 to 1000, was assigned.

### Patient samples and cell fractionation

After obtaining The University of Texas MD Anderson Cancer Center Institutional Review Board-approved informed consent, we obtained peripheral blood (PB) cells from 3 patients with CLL. To isolate low-density cells, we fractionated PB cells using Ficoll Hypaque 1077 (Sigma-Aldrich). More than 90% of the CLL PB cells were CD19+/CD5+ lymphocytes.

### Generation of green fluorescence protein (GFP)-lentiviral STAT3-shRNA and infection of CLL cells

293T cells were co-transfected with GFP-lentivirus STAT3 short hairpin RNA (shRNA) or GFP-lentivirus empty vector and the packaging vectors pCMVdeltaR8.2 and pMDG (generously provided by Dr. G. Inghirami, Torino, Italy) using the Superfect transfection reagent (Qiagen, Inc.). 293T cell culture medium was changed after 16 hours and collected after 48 hours. The culture medium was filtered through a 45-μm syringe filter to remove floating cells, the lentivirus was then concentrated by filtration through an Amicon ultracentrifugal filter device (Millipore, Billerica, MA), and the concentrated supernatant was used to infect CLL cells. CLL cells (5 × 10^6^/mL) were incubated in 6-well plates (Becton Dickinson, Franklin Lakes, NJ) in 2 ml DMEM supplemented with 10% fetal calf serum and transfected with 100 μL of viral supernatant. Polybrene (10 ng/mL) was added to the viral supernatant at a ratio of 1:1000 (v/v). Infection efficiency was measured after 48–72 hours and was found to range between 40% and 70% (calculated on the basis of the ratio of propidium iodide (PI)-negative/GFP-positive cells).

### RNA purification and quantitative real-time polymerase chain reaction (RT-PCR)

Total RNA was extracted using Trizol reagent (Invitrogen) for microRNA analyses according to the manufacturer's protocol. For mature microRNA expression analysis, total RNA was retrotranscribed with microRNA-specific primers using TaqMan microRNA reverse transcription kit (Applied Biosystems, Foster City, CA), and then quantitative RT-PCR was performed using Taqman miR assays according to the manufacturer's protocol. The comparative cycle time (Ct) method was used to calculate the relative abundance of microRNAs compared with snRNA U6 as an internal control for RNA normalization. The profiling was done in duplicate wells for each sample and in two independent experiments (three measurements each), and the results were presented as mean +/− SD of the four measurements.

### Western immunoblotting

Western blot analysis was conducted using mouse anti-human STAT3 antibodies (BD Bioscience, Palo Alto, CA) and horseradish peroxidase-conjugated anti-mouse secondary antibodies (GE, healthcare, Amersham, Buckinghamshire, UK), as previously described [4].

### Microarray analysis

Microarray analysis was conducted to identify miRs that were differentially expressed after silencing of STAT3 by transfection with short hairpin (sh) RNA or control treatment by transfection with empty vector. RNA was labeled and hybridized on miR microarray chips as previously described [13]. Signals in the images were quantified with GenePix Pro 6.0 software (Axon Instruments), and data were analyzed using PartekFlow software (v 2.1, 2012). Following quantile normalization, expression differences between the two sets of transfected CLL cells were tested by application of the Welch’s approximate *t*-test for two groups (with variances not assumed equal), with a *P* value cutoff of 0.05. Hierarchical clustering was generated for both genes and conditions, by using standard correlation as a measure of similarity.

### Bioinformatics tools

The RNA22 pattern discovery algorithm, which utilizes miR sequences to predict miR:mRNA heteroduplexes, was used to simulate the sponge-out process [14].

## Results and discussion

### Putative STAT3 binding sites exist in the promoters of 25% of miR genes

A histone modification in which histone H3 is trimethylated on lysine 4 residues (H2K4me3) typifies transcription start sites in most gene promoters [15]. To identify miR gene promoters, Baer et al. interrogated these regions in CLL cells, normal B cells, and CLL-related cell lines [11]. Among the 939 miRs annotated to the miRBase15 database [16], they identified putative miR promoters in 781 miR genes [11].

Using those reports’ and the ChIP-seq ENCODE’s data [12], we searched for STAT3-binding sites in miR promoters. In our search, we identified 52,348 plausible STAT3-binding sites; STAT3 binding sites were found in 160 putative promoters of nearly 25% of the miR genes examined (N=200) with binding scores ranging from 100 (the lowest score) to 1000 (the highest score). Binding sites were found in 132 promoters of single pre-miR genes and in 28 promoters of pre-miR clusters of 2 to 6 genes (Table 1 and Additional file 2: Table S1). High STAT3-binding scores of 1000 and 710 were identified in miR-21 and miR-181b, consistent with the data of Iliopoulos et al. [9]. Remarkably, putative STAT3-binding sites were also detected in the promoters of miR-15-1 and miR-16a, which are deleted in most CLL patients with del(13q) [17].

**Table 1:** MiRs with putative STAT3 binding sites

**Micro RNA gene**	**Chromosome**	**Promoter start coordinates**	**Promoter end coordinates**	Median (range) STAT3 binding score*
miR-1205, miR-1206,miR-1207	8 q24.21	128961454	128962791	1000 (1000-1000)
miR-1537	1 q42.3	236045425	236047415	1000 (1000-1000)
miR-21	17 q23.1	57901872	57921277	1000 (112-1000)
miR-3124	1 q44	249115404	249123965	1000 (1000-1000)
miR-451	17q11.2	27222251	27224114	1000 (1000-1000)
miR-92b	1 q22	155162340	155168439	1000 (1000-1000)
miR-3197	21 q22.2	42537544	42543023	943 (943-943)
miR-646	20 q13.33	58712550	58715320	789 (789-789)
miR-629	15 q23	70383751	70394586	773 (661-885)
miR-30e, miR-30c-1	1 p34.2	41173077	41177703	759 (759-759)
miR-3125	2 p24.3	12855381	12862915	756 (756-756)
miR-3145	6 q23.3	138776942	138779365	743 (487-1000)
miR-645	20 q13.13	49199911	49201187	743 (743-743)
miR-1256	1 p36.12	21346830	21350211	725 (725-725)
miR-619	12 q24.11	109248263	109253306	719 (719-719)
miR-181a-2, miR-181b-2	9 q33.3	127418928	127426139	710 (710-710)
miR-29a, miR-29b-1	7 q32.3	130583383	130597803	697 (482-1000)
miR-202	10 q26.3	135069499	135077337	696 (393-1000)
miR-3142, miR-146a	5 q34	159890882	159899475	671 (671-671)
miR-548c	12 q14.2	65000968	65011503	660 (660-660)
miR-630	15 q24.1	72764289	72769197	627 (255-1000)
miR-135b	1 q32.1	205416952	205452990	622 (245-1000)
miR-29c, miR-29b-2	1 q32.2	207991044	208002382	608 (608-608)
miR-1825	20 q11.21	30791020	30798310	604 (209-1000)
miR-548h-1	14 q23.2	64578834	64581657	587 (174-1000)
miR-612	11 q13.1	65183633	65198528	581 (157-1000)
miR-148b	12 q13.13	54717640	54721204	578 (578-578)
miR-3174	15 q26.1	90543381	90549092	576 (152-1000)
let7a-3, let7b	22	46480680	46481826	573 (146-1000)
miR-1255a	4 q24	102263848	102272541	557 (557-557)

*MiRs with the highest STAT3 binding score obtained by using the ENCODE ChIP-seq data (Pennisi 2012) [7] are depicted.

### STAT3 upregulates miR levels in CLL cells

**Figure 1:**
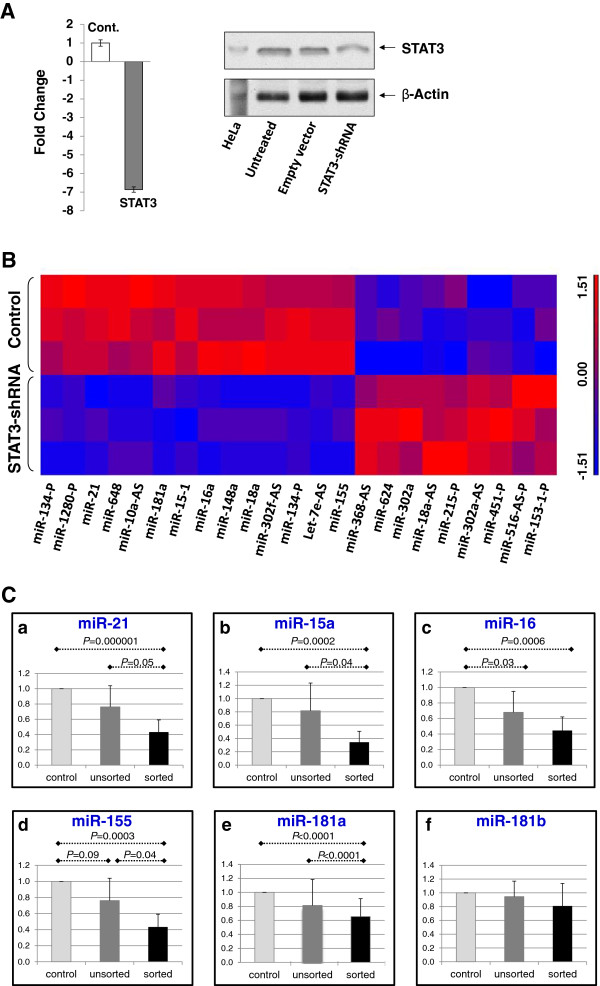
**Transfection with STAT3-shRNA alters miR gene expression in CLL cells. A.** Left panel: Quantitative RT-PCR shows an 8 fold reduction in STAT3 mRNA levels in CLL cells transfected with STAT3-shRNA compared to control CLL cells transfected with an empty vector. The means ± S.D. of 3 experiments are depicted. Right panel: Western immunobloting shows a decrease in STAT3 protein levels of CLL cells transfected with STAT3-shRNA compared to untreated cells or cells transfected with an empty vector. **B.** MiR array heat map depicts 23 miRs whose expression differed significantly between CLL cells transfected with STAT3-shRNA and CLL cells transfected with empty vector. Red, high levels; blue, low levels. **C.** Panels **a** through **f** show the results of RT-PCR analysis of CLL cells transfected with STAT3-shRNA. The plots show relative expression as means ± SD of 3 replicates for each miR tested. Shown are relative expression of miRs in the unsorted STAT3-shRNA transfected CLL cells (transfection efficiency: 30%; middle, gray bars), sorted STAT3-shRNA-transfected CLL cells (black bars), and empty vector-transfected cells (light gray bars labeled “control”).

Because putative STAT3 binding sites were identified in a significant number of miR promoters and STAT3 is constitutively activated in CLL, we sought to determine whether STAT3 would affect miR levels in unstimulated PB CLL cells. Transfection of CLL with STAT3-shRNA downregulated STAT3-mRNA and protein levels (Figure 1A). Microarray analysis showed that RNA levels of STAT3-shRNA-transfected cells were significantly different (*P* ≤ 0.05) from RNA levels of empty vector-transfected cells in 152 probes from 78 non-coding RNA genes, including 72 miR genes; the levels of 63 of the 72 miR genes were significantly downregulated by STAT3-shRNA, suggesting that STAT3 upregulates the levels of those miRs (Additional file 3: Table S2). For 60% of the 63 downregulated miR genes (n = 38) ChIP-seq data confirmed STAT3 binding in a putative promoter upstream of the gene location, significantly more than expected by chance (p<0.0001) (Additional file 2: Table S1).

To confirm these data, we studied miR levels of CLL cells transfected with STAT3-shRNA or empty vector using RT-PCR. Analyzed were miRs with either high levels of STAT3-binding sites, as reported in the ChIP-seq database (i.e. miR-21), reduced STAT3 levels following transfection with STAT3-shRNA in our studies (miR-181a), high STAT3-binding scores (213 for miR15-1 and miR-16a and 1,000 for miR-21), or a known pathogenetic role in CLL (miR15-1, miR-16a, miR-155, and miR-21) [18-20]. As shown in Figure 1B, RT-PCR confirmed the array data in 5 of the 6 studied miRs.

### STAT3 downregulates miR levels in CLL cells

Phosphorylated STAT3 binds to and activates the STAT3 gene, inducing the production of STAT3 protein [4]. Our current analysis revealed that STAT3 binds several miRs whose levels are overexpressed in CLL such as *miR-21*, an oncomiR of various neoplasms [20], and *miR-29* and *-181a,* which affect CLL cell survival [21]. The levels of most miRs we studied were downregulated by transfection with STAT3-shRNA; however, the levels of 9 miRs were upregulated (Figure 1; Additional file 3: Table S2), suggesting that STAT3 downregulates those miRs’ levels. Several pathways can lead to STAT3 mediated suppression of transcription. Direct binding of unphosphorylated STAT3 suppresses transcription [22]. For example, miR-451, whose levels are downregulated by STAT3, carries a very high STAT3 binding score in its promoter. In addition, STAT3 mediates epigenetic silencing of a variety of genes [23], in particular genes that affect histone deacetylases [24,25]. Therefore, we theorized that STAT3 induces epigenetic silencing of various miRs in CLL cells. Finally, STAT3 may also operate in a transcription-independent fashion. Single-stranded RNAs were shown to sequester (“sponge”) miRs in a sequence-specific manner [26]. Because constitutively phosphorylated STAT3 activates the STAT3 gene, single-stranded 4.3-kb STAT3-mRNA is generated at high levels in CLL cells. Those 4.3-kb RNA strands may bind complementary miRs, thereby interrupting their interaction with their corresponding target genes. For example, miR-18-a antisense (AS), upregulated by STAT3-shRNA has a complementary sequence in STAT3 mRNA, consistent with the “sponge regulation” hypothesis. Conversely, the miR-18-a sense form, downregulated by STAT3-shRNA, does not have a complementary sequence in STAT3 or the 3’UTR-STAT3.

To test whether this mechanism may be operative in CLL cells, we performed a simulation analysis using the 9 miRs whose levels were upregulated by STAT3-shRNA. We compared the theoretical energy released by single-stranded STAT3 mRNA sequences to that of random 4.3-kb mRNA sequences with an identical base content. Because miRs preferentially bind 3’-untranslated region sequences, we also calculated the energy release of shorter mRNA sequences. Using the RNA22 algorithm, we found higher energy release by full-length and short 3’-untranslated region STAT3 mRNA than by random single-stranded RNA sequences, suggesting that complementary binding of single-stranded STAT3 mRNA to miRs sequestered the circulating miRs, generating a stable, energetically superior state (Additional file 3: Table S2).

Taken together, our data suggest that STAT3 directly and indirectly modulates miR expression in CLL cells. Further studies to explore the mechanisms that affect miR expression in CLL are warranted.

## Competing interests

The authors declare that they have no competing interests.

## Authors’ contributions

UR drafted the manuscript and performed the bioinformatics and statistical analysis, GC conceived and designed, T S performed the RT-PCR experiments, L A performed the RT-PCR, DM performed the sh-RNA experiment, PL helped in sh-RNA experiment, ZL helped in sh-RNA experiment, SG performed part of the RT-PCR analysis, AF recruited patients to the study, JB obtained patients’ samples, SO obtained patients’ samples, WW recruited patients to the study, MK participated in designing ZE conceived, designed and helped to draft the manuscript. All authors read and approved the final manuscript.

## Supplementary Material

Additional file 1 Genomic coordinates of STAT3 in miRs genes promoters. Click here for file

Additional file 2: Table S1 List of 160 miRs whose promoters harbor putative STAT3 binding sites. Click here for file

Additional file 3: Table S2 Simulation of energy released from STAT3 true single-stranded RNA and random RNA sequences in 9 miRs upregulated by STAT3-shRNA. Click here for file
