# Supplementary material for: Signal transducer and activator of transcription (STAT)-3 regulates microRNA gene expression in chronic lymphocytic leukemia cells
Source: Mol Cancer. 2013 Jun 1;12:50. doi: 10.1186/1476-4598-12-50 (PMC3671957; doi:10.1186/1476-4598-12-50)
Supplement: Additional file 1 — Genomic coordinates of STAT3 in miRs genes promoters. [file 1476-4598-12-50-S1.pptx]

## Slide 1
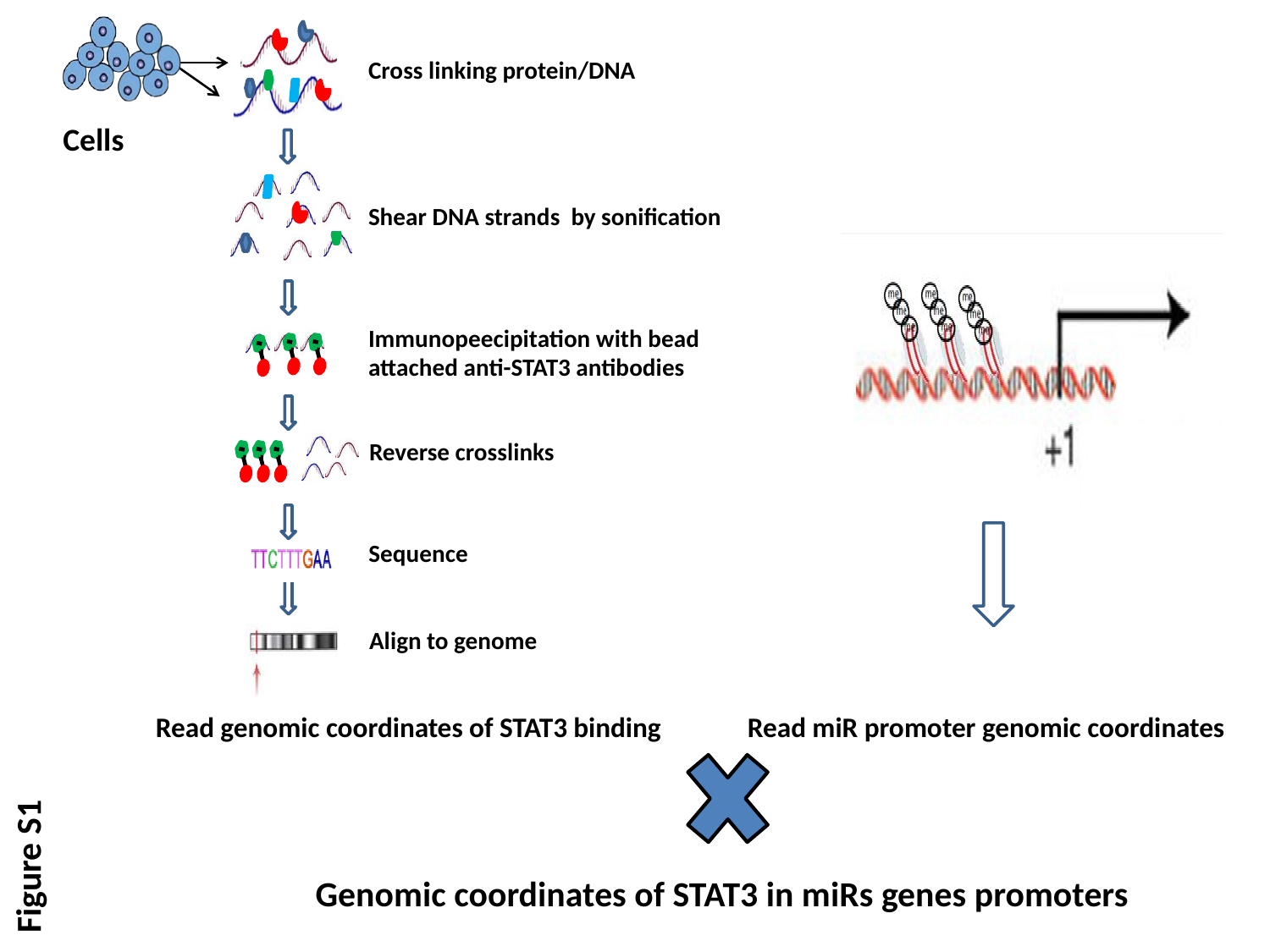

Cross linking protein/DNA
Cells
Shear DNA strands by sonification
Immunopeecipitation with bead attached anti-STAT3 antibodies
Reverse crosslinks
Sequence
Align to genome
Read genomic coordinates of STAT3 binding
Read miR promoter genomic coordinates
Figure S1
Genomic coordinates of STAT3 in miRs genes promoters
