# Supplementary material for: Signal transducer and activator of transcription (STAT)-3 regulates microRNA gene expression in chronic lymphocytic leukemia cells
Source: Mol Cancer. 2013 Jun 1;12:50. doi: 10.1186/1476-4598-12-50 (PMC3671957; doi:10.1186/1476-4598-12-50)
Supplement: Additional file 2: Table S1 — List of 160 miRs whose promoters harbor putative STAT3 binding sites. [file 1476-4598-12-50-S2.docx]

**Table S1. List of 160 miRs whose promoters harbor putative STAT3 binding sites**

| Micro RNA gene | Chromosome | Promoter start coordinates | Promoter end coordinates | Median (range) STAT3 binding score* |
| --- | --- | --- | --- | --- |
| miR-1205, miR-1206, miR1207 | 8 q24.21 | 128961454 | 128962791 | 1000 (1000-1000) |
| miR-1537 | 1 q42.3 | 236045425 | 236047415 | 1000 (1000-1000) |
| **miR-21** | **17 q23.1** | **57901872** | **57921277** | **1000 (112-1000)** |
| miR-3124 | 1 q44 | 249115404 | 249123965 | 1000 (1000-1000) |
| **miR-451** | 17q11.2 | 27222251 | 27224114 | 1000 (1000-1000) |
| miR-92b | 1 q22 | 155162340 | 155168439 | 1000 (1000-1000) |
| miR-3197 | 21 q22.2 | 42537544 | 42543023 | 943 (943-943) |
| miR-646 | 20 q13.33 | 58712550 | 58715320 | 789 (789-789) |
| miR-629 | 15 q23 | 70383751 | 70394586 | 773 (661-885) |
| miR-30e, miR-30c-1 | 1 p34.2 | 41173077 | 41177703 | 759 (759-759) |
| miR-3125 | 2 p24.3 | 12855381 | 12862915 | 756 (756-756) |
| miR-3145 | 6 q23.3 | 138776942 | 138779365 | 743 (487-1000) |
| miR-645 | 20 q13.13 | 49199911 | 49201187 | 743 (743-743) |
| miR-1256 | 1 p36.12 | 21346830 | 21350211 | 725 (725-725) |
| miR-619 | 12 q24.11 | 109248263 | 109253306 | 719 (719-719) |
| **miR-181a-2,** **miR-181b-2** | 9 q33.3 | 127418928 | 127426139 | 710 (710-710) |
| miR-29a, miR-29b-1 | 7 q32.3 | 130583383 | 130597803 | 697 (482-1000) |
| **miR-202** | 10 q26.3 | 135069499 | 135077337 | 696 (393-1000) |
| miR-3142, miR-146a | 5 q34 | 159890882 | 159899475 | 671 (671-671) |
| miR-548c | 12 q14.2 | 65000968 | 65011503 | 660 (660-660) |
| miR-630 | 15 q24.1 | 72764289 | 72769197 | 627 (255-1000) |
| miR-135b | 1 q32.1 | 205416952 | 205452990 | 622 (245-1000) |
| miR-29c, miR-29b-2 | 1 q32.2 | 207991044 | 208002382 | 608 (608-608) |
| **miR-1825** | 20 q11.21 | 30791020 | 30798310 | 604 (209-1000) |
| miR-548h-1 | 14 q23.2 | 64578834 | 64581657 | 587 (174-1000) |
| miR-612 | 11 q13.1 | 65183633 | 65198528 | 581 (157-1000) |
| miR-148b | 12 q13.13 | 54717640 | 54721204 | 578 (578-578) |
| miR-3174 | 15 q26.1 | 90543381 | 90549092 | 576 (152-1000) |
| **let7a-3, let7b** | **22** | **46480680** | **46481826** | **573 (146-1000)** |
| miR-1255a | 4 q24 | 102263848 | 102272541 | 557 (557-557) |
| miR-31 | 9 p21.3 | 21506583 | 21520921 | 551 (261-842) |
| miR-614 | 12 p13.1 | 13042649 | 13049659 | 546 (336-757) |
| miR-1193, miR-543, miR-495 | 14[q32.31](http://www.ensembl.org/Homo_sapiens/contigview?chr=14&band=q32.31) | 101468007 | 101470695 | 526 (526-526) |
| miR-1259 | 20* | 47891243 | 47898128 | 517 (517-517) |
| miR-146b | 10 q24.32 | 104189728 | 104197915 | 516 (516-516) |
| miR-3138 | 4 p16.1 | 10108444 | 10109686 | 515 (515-515) |
| miR-4323 | 19 q13.2 | 42632112 | 42639979 | 511 (511-511) |
| miR-28 | 3 q28 | 187868975 | 187873972 | 509 (509-509) |
| miR-3122 | 1 q32.3 | 212206390 | 212211151 | 507 (225-789) |
| miR-1231 | 1 q32.1 | 201706457 | 201711479 | 488 (488-488) |
| miR-3175 | 15 q26.1 | 93440753 | 93453207 | 484 (378-846) |
| miR-563 | 3 p25.1 | 15901235 | 15904054 | 480 (480-480) |
| miR-940 | 16 p13.3 | 2314034 | 2322243 | 476 (476-476) |
| miR-550-1 | 3 p21.31 | 30322442 | 30329470 | 474 (256-693) |
| miR-1226 | 7 q32.1 | 47841910 | 47847182 | 468 (468-468) |
| miR-593 | 16 q24.1 | 127290815 | 127294730 | 458 (458-458) |
| miR-1910 | 10 q24.32 | 85782186 | 85786608 | 448 (448-448) |
| miR-4259 | 1q23.2 | 159889862 | 159898336 | 430 (124-737) |
| miR-3159 | 11 p15.1 | 18404788 | 18406867 | 429 (429-429) |
| **miR-324** | **17 p13.1** | **7132650** | **7148312** | **404 (404-404)** |
| miR-637 | 19 p13.3 | 3968186 | 3972184 | 399 (179-620) |
| miR-933 | 2 q31.1 | 176028350 | 176036318 | 398 (398-398) |
| miR-3168 | 13 q14.11 | 41700490 | 41708600 | 391 (391-391) |
| **miR-155** | **21 q21.3** | **26932079** | **26947820** | **383 (383-383)** |
| miR-224, miR-452 | Xq28 | 151132391 | 151139396 | 379 (181-578) |
| miR-198 | 3q13.33 | 120135804 | 120137956 | 375 (375-375) |
| miR-641 | 19 q13.2 | 40783225 | 40793171 | 367 (367-367) |
| miR-548s | 2 p25.1 | 11883772 | 11896030 | 366 (336-431) |
| miR-548h-3 | 17p12 | 13446967 | 13448590 | 361 (361-361) |
| miR-99a, let-7c, miR-125b | 21 q21.1 | 17564208 | 17569306 | 348 (348-348) |
| miR-2909 | 17 q12 | 35303759 | 35308670 | 347 (347-347) |
| miR-567 | 3 q13.2 | 111803095 | 111808273 | 346 (346-346) |
| miR-484 | 16 p13.11 | 15724567 | 15740605 | 345 (114-571) |
| miR-1244-1 | 2q37.1 | 232569371 | 232578029 | 337 (337-337) |
| miR-580 | 5 p13.2 | 36147450 | 36154533 | 335 (335-335) |
| miR-4262 | 2 p25.1 | 12004143 | 12006504 | 333 (333-333) |
| miR-3143 | 6 p22.1 | 27097082 | 27117866 | 316 (184-332) |
| miR-3166 | 11 q14.2 | 87905898 | 87909816 | 306 (306-306) |
| miR-571 | 4 p16.3 | 329068 | 333818 | 304 (304-304) |
| **miR-375** | **2 q35** | **219860844** | **219863705** | **299 (299-299)** |
| miR-603 | 10 p12.2 | 24496203 | 24529077 | 298 (181-415) |
| **miR-1304** | 11 q21 | 93468360 | 93476502 | 292 (246-339) |
| miR-3128 | 2 q31.2 | 178124217 | 178132103 | 289 (198-380) |
| **miR-95** | 4 p16.1 | 8030233 | 8031472 | 283 (283-283) |
| miR-1305 | 4 q34.3 | 183058618 | 183065713 | 272 (272-272) |
| let7g | 17 p13.3 | 52302628 | 52315178 | 268 (159-377) |
| miR-22 | 16 p13.3 | 1615837 | 1622260 | 268 (227-310) |
| miR-3176 | 2 q37.3 | 574074 | 577665 | 267 (267-267) |
| miR-4269 | 19 p13.13 | 240195177 | 240199755 | 267 (267-267) |
| **miR-24-2,** **miR-27a**,miR-23a | 19 p13.13 | 13948858 | 13960268 | 263 (109-856) |
| miR-554 | 8 p22 | 151511617 | 151516474 | 262 (124-326) |
| miR-548v | 8 p11.21 | 17547882 | 17551448 | 260 (260-260) |
| miR-486 | 2 q35 | 41541449 | 41542592 | 258 (258-258) |
| miR-190 | 15q22.2 | 63033814 | 63035144 | 257 (257-257) |
| **miR-23b**, miR-27b, **miR-24-1** | **9 q22.32** | **97763989** | **97769651** | **256 (256-256)** |
| miR-150 | 19 q13.33 | 49999110 | 50004613 | 255 (255-255) |
| **miR-423**, **miR-3184** | **17 q11.2** | **28442062** | **28446209** | **254 (246-262)** |
| **miR-575** | 4 q21.22 | 83705649 | 83708153 | 251 (251-251) |
| miR-4266 | 2q12.3 | 109947977 | 109953156 | 250 (192-308) |
| miR-3198 | 22q11.21 | 18251344 | 18266069 | 246 (246-246) |
| miR-936 | 10q25.1 | 105836246 | 105837477 | 246 (246-246) |
| miR-3150 | 8q22.1 | 96035914 | 96039473 | 245 (245-245) |
| miR-483 | 11 p15.5 | 2160810 | 2168940 | 238 (238-238) |
| miR-197 | 1 p13.3 | 110088712 | 110093658 | 237 (237-237) |
| miR-628 | 15 q21.3 | 55697947 | 55700680 | 236 (236-236) |
| miR-15b, miR-16-2 | 3 q25.33 | 160113196 | 160124595 | 234 (234-234) |
| miR-7-1 | 9 q21.32 | 86590163 | 86599227 | 233 (233-233) |
| miR-1237 | 11 q13.1 | 64125268 | 64129210 | 231 (231-231) |
| miR-3193 | 20 q11.21 | 30192581 | 30196823 | 231 (148-315) |
| miR-4280 | 5 q14.3 | 86416340 | 86419610 | 230 (230-230) |
| miR-616 | 12 q13.3 | 57912757 | 57920056 | 228 (228-228) |
| miR-450b, miR-450a-1, miR-450a-2,**miR-542**, miR-503, miR-424 | **q26.3** | **133676422** | **133687904** | **223 (223-223)** |
| miR-4263 | 2 p23.2 | 28110283 | 28116569 | 222 (222-222) |
| miR-708 | 11 q14.1 | 79142357 | 79143458 | 213 (213-213) |
| miR-3153 | 9 q22.2 | 91924220 | 91932725 | 212 (212-212) |
| miR-658, miR-659 | 22 q13.1 | 38243249 | 38245716 | 211 (211-211) |
| miR-661 | 8 q24.3 | 145014042 | 145028899 | 207 (128-312) |
| miR-1260b | 11 q21 | 96069409 | 96077345 | 206 (206-206) |
| miR-1243 | 4 | 113737264 | 113741541 | 195 (195-195) |
| miR-3182 | 16 q23.3 | 82659539 | 82662195 | 195 (195-195) |
| miR-4258 | 1 q21.3 | 154940212 | 154950946 | 194 (116-1000) |
| miR-3157 | 10 q24.1 | 97846439 | 97859661 | 189 (189-189) |
| **miR-876** | **9 p21.1** | **28883931** | **28888014** | **189 (189-189)** |
| miR-504 | q26.3 | 137770286 | 137777597 | 188 (188-188) |
| miR-576 | 4 q25 | 110352854 | 110357400 | 187 (187-187) |
| **miR-15a, miR-16-1** | 13 q14.2 | 50645357 | 50658720 | 183 (153-213) |
| **miR-620** | 12 q24.21 | 116589979 | 116591190 | 183 (183-183) |
| miR-1255b-2 | 1q24.2 | 167903382 | 167908920 | 182 (118-246) |
| miR-1975* | 7 | 148635972 | 148640892 | 180 (180-180) |
| miR-635 | 17 q24.2 | 66450332 | 66456209 | 179 (179-179) |
| **miR-221, miR-222** | **p11.3** | **45613300** | **45636390** | **177 (149-191)** |
| miR-1179, **miR-7-2** | **15 q26.1** | **89145881** | **89151993** | **170 (170-170)** |
| miR-103-2 | 20p13 | 3864752 | 3877209 | 169 (169-169) |
| miR-125b-2 | 21q21.1 | 17943322 | 17960804 | 168 (106-230) |
| miR-1976 | 1 p36.11 | 26854520 | 26876649 | 168 (167-170) |
| miR-935 | 19 q13.42 | 54478564 | 54487106 | 167 (167-167) |
| miR-548p | 5 q21.1 | 100175833 | 100178610 | 164 (164-164) |
| miR-1278 | 1 q31.2 | 193088666 | 193094873 | 163 (163-163) |
| miR-320e | 19 q13.32 | 47216339 | 47224058 | 163 (163-163) |
| miR-640 | 19 p13.11 | 19513827 | 19520124 | 162 (162-162) |
| miR-891a | Xq27.3 | 145114506 | 145140790 | 161 (116-490) |
| let-7i | 12 | 62994117 | 63000945 | 160 (160-160) |
| miR-1284 | 3 p13 | 71612747 | 71614482 | 158 (158-158) |
| miR-34a | 1 p36.22 | 9223246 | 9225404 | 153 (153-153) |
| miR-548e | 10 q25.2 | 112676814 | 112681779 | 153 (138-168) |
| miR-615 | 12 q13.13 | 54425620 | 54433298 | 153 (153-153) |
| miR-195, miR-497 | 17 p13.1 | 6913318 | 6928634 | 150 (150-150) |
| miR-205 | 1 q32.2 | 209602229 | 209603515 | 145 (145-145) |
| miR-3137 | 3 q29 | 194867289 | 194873594 | 145 (145-145) |
| miR-3191, miR-3190 | 19q13.32 | 47729942 | 47734039 | 138 (137-140) |
| miR-2110 | 10 q25.3 | 115930237 | 115935592 | 135 (102-168) |
| miR-1289-1 | 20 q11.22 | 34040783 | 34045499 | 134 (134-134) |
| miR-1204, miR-1205, miR-1206, miR1207 | 8q24.21 | 128805019 | 128813419 | 131 (122-140) |
| miR-556 | 1 q23.3 | 162037115 | 162281329 | 129 (106-152) |
| **miR-624** | 14 q12 | 31491137 | 31498899 | 129 (129-129) |
| miR-365-2 | 17 q11.2 | 29894990 | 29896427 | 122 (122-122) |
| **miR-569** | **3 q26.2** | **170833862** | **170835052** | **120 (120-120)** |
| miR-590 | 7 q11.23 | 73601510 | 73602669 | 120 (120-120) |
| miR-1236 | 6 p21.33 | 31924259 | 31929518 | 115 (115-115) |
| miR-101-1, miR-3671 | 1 p31.3 | 65547864 | 65549998 | 113 (113-113) |
| miR-1181 | 19 p13.2 | 10511646 | 10518668 | 113 (113-113) |
| miR-193b, miR-365-1 | 16 p13.12 | 14393598 | 14405741 | 113 (113-113) |
| **let7a-1, let7f-1, let7d** | 9 | 96926171 | 96936157 | 112 (107-118) |
| **miR-3173** | 14 q32.13 | 95619109 | 95626341 | 112 (112-112) |
| miR-320a | 8 p21.3 | 22102481 | 22105775 | 111 (111-111) |
| **miR-1302-1** | 12 q24.13 | 113143102 | 113144920 | 108 (108-108) |
| **miR-133a-1, miR-1-2** | 18q11.2 | 19404698 | 19409921 | 108 (108-108) |
| miR-491 | 9 p21.3 | 20680984 | 20688366 | 108 (104-113) |
| miR-545, **miR-374a** | **X q13.2** | **73506379** | **73515417** | **100 (100-100)** |

MiRs with the highest STAT3 bindings scores according to the ENCODE ChIP-seq database are depicted. Highlighted are the miRs whose levels were downregulated by transfection with STAT3-shRNA.

*MiR-1259 is not included in the miRbase 19 version
