# Supplementary material for: Signal transducer and activator of transcription (STAT)-3 regulates microRNA gene expression in chronic lymphocytic leukemia cells
Source: Mol Cancer. 2013 Jun 1;12:50. doi: 10.1186/1476-4598-12-50 (PMC3671957; doi:10.1186/1476-4598-12-50)
Supplement: Additional file 3: Table S2 — Simulation of energy released from STAT3 true single-stranded RNA and random RNA sequences in 9 miRs upregulated by STAT3-shRNA. [file 1476-4598-12-50-S3.docx]

**Table S2. Simulation of energy released from STAT3 true single-stranded RNA and random RNA sequences in 9 miRs upregulated by STAT3-shRNA**

| **STAT3 element of interest** | RNA sequence | Number of miRs with binding sites/number of miRs analyzed | Total number of predicted miR binding sites | Theoretical energy released from miR binding (Kcal/mol) |
| --- | --- | --- | --- | --- |
| STAT3 | True | 6/9 | 111 | 2458 |
|  | Random | 7/9 | 146 | 1627 |
| STAT3 3’-untranslated region | True | 6/9 | 52 | 1087 |
|  | Random | 5/9 | 36 | 671 |
| STAT3+STAT3 3’-untranslated region | True | 12/18 | 163 | 3536 |
|  | Random | 12/18 | 147 | 2298 |
